# Inducers and Inhibitors of Pyroptotic Death of Granulosa Cells in Models of Premature Ovarian Insufficiency and Polycystic Ovary Syndrome

**DOI:** 10.1007/s43032-024-01643-3

**Published:** 2024-07-18

**Authors:** Caglar Berkel

**Affiliations:** Department of Molecular Biology and Genetics, Tokat Gaziosmanpasa University, Tokat, Türkiye

**Keywords:** Caspase 1, Cell death, Granulosa cells, GSDMD, Interleukin, NLRP3, Polycystic Ovary Syndrome, Premature Ovarian Insufficiency, Pyroptosis

## Abstract

Granulosa cells (GCs), the largest cell population and primary source of steroid hormones in the ovary, are the important somatic ovarian components. They have critical roles in folliculogenesis by supporting oocyte, facilitating its growth, and providing a microenvironment suitable for follicular development and oocyte maturation, thus having essential functions in maintaining female fertility and in reproductive health in general. Pyroptotic death of GCs and associated inflammation have been implicated in the pathogenesis of several reproductive disorders in females including Premature Ovarian Insufficiency (POI) and Polycystic Ovary Syndrome (PCOS). Here, I reviewed factors, either intrinsic or extrinsic, that induce or inhibit pyroptosis in GCs in various models of these disorders, both in vitro and in vivo, and also covered associated molecular mechanisms. Most of these studied factors influence NLRP3 inflammasome- and GSDMD (Gasdermin D)-mediated pyroptosis in GCs, compared to other inflammasomes and gasdermins (GSDMs). I conclude that a more complete mechanistic understanding of these factors in terms of GC pyroptosis is required to be able to develop novel strategies targeting inflammatory cell death in the ovary.

## Introduction

Female subfertility is an increasing reproductive issue worldwide, which is in part due to abnormal ovarian follicular development, affecting one in six couples clinically. The number and the quality of follicles in the ovary is highly related with the fertility status in women [40, 41, 45]. Each follicle consists of an oocyte surrounded by many granulosa cells (GCs). These cells support oocyte, facilitate its growth, and provide a suitable microenvironment for follicular development and oocyte maturation. Although granulosa cell death can be initiated by other programmed cell death pathways such as apoptosis and necroptosis upon certain stimuli [117], here, I focused on pyroptotic death of granulosa cells, factors that induce or limit pyroptosis of these cells, and their implications in reproductive diseases such as Premature Ovarian Insufficiency (POI) and Polycystic Ovary Syndrome (PCOS).

Some transgender men and other gender-diverse people can also experience these disorders; however, since most of the previous work refers to people experiencing them collectively as “women” and does not particularly clarify how these findings might apply to the specific needs of gender-diverse people, I have also used “women” in certain instances, to avoid inappropriate generalisation [7, 9, 36, 50, 59]. More research is urgently needed about the experience of Premature Ovarian Insufficiency (POI) and Polycystic Ovary Syndrome (PCOS) in transgender men and gender-diverse people. Evidence on these disorders in these groups of people is highly scarce, and this research area needs more scientific attention.

### Granulosa Cells (GCs)

The development of ovarian follicles capable of ovulating and releasing a mature fertilizable oocyte is a highly complex process that is regulated by both intrafollicular factors and peripheral hormones, including follicle stimulating hormone (FSH). Follicular development can be divided into several stages, including primordial, primary, secondary, and tertiary (antral) follicles [8]. Follicles are structures of critical importance in the ovary, with each follicle consisting of an oocyte surrounded by granulosa cells (GCs). GCs, the largest cell group and primary steroidogenic source in the ovary, are the important somatic cells in the ovary, and play important roles in folliculogenesis by supporting oocyte, facilitating oocyte growth, and providing a suitable microenvironment for follicular development and oocyte maturation, thus having essential functions in maintaining female fertility. GCs primarily secrete multiple growth factors and hormones (essential reproductive hormones such as estrogen and progesterone) that interact with the oocyte during folliculogenesis which is the process of ovarian follicle development from primordial germ cells to fertilizable mature oocytes [82, 153]. The direct communication in addition to the paracrine interactions between the oocyte and GCs provide essential metabolic support for the development and maturation of the oocyte [118]. During ovarian follicular development, oocytes gradually increase in size and progress to maturation accompanied by the proliferation and progressive differentiation of the surrounding somatic cells, including GCs and theca cells (TCs), to support oocyte maturation and ovulation [87]. This proliferation and differentiation of GCs are key events during follicle development, which is regulated by complex communication and interaction among oocytes, GCs, TCs and other somatic cells of the ovary.

There are two major types of GCs: cumulus cells and mural GCs, which surround the oocytes and the antrum (part of an ovarian follicle filled with follicular fluid), respectively. Cumulus cells are involved in providing necessary nutrients to the oocyte and in influencing the development of the oocyte, while mural GCs have endocrine functions and support the growth of the follicle [116]. Estrogens including estradiol and estrone are the key hormones produced by GCs in response to FSH and to the diffusion of androgens from theca cells. Besides, GCs produce different growth factors which interact with the oocyte during its development and impact the process of follicular growth [125, 146]. Therefore, dysfunction of GCs results in abnormal follicular development and ovulation, and ultimately compromises female fertility [80].

Primordial follicles are established perinatally, and they are non-growing (i.e. remain quiescent until activated) and consist of a central primary oocyte surrounded by a single layer of flattened pre-granulosa cells (pre-GCs) [48, 120]. For the females of mammalian species, the pool of primordial follicles provides developing follicles and fertilizable ova resources throughout the reproductive lifespan [2, 148]. In each cycle, only a small number of primordial follicles are selectively activated through a process termed primordial follicle activation. Once activated, primordial follicles undergo rapid growth, resulting in the enlargement of oocytes and proliferation of GCs [84]. During this process, GCs particularly show the most apparent morphological changes, from monolayer flattened to cuboidal in shape. Accordingly, GCs are accompanied by a transition from a relatively transcriptionally inactive state to a more active state [149]. Extensive research have focused on the molecular interactions between oocytes and the surrounding GCs, and this bidirectional signaling has been shown to be required for the proper control of primordial follicle activation, proper oocyte maturation and reproductive health in general [21, 98].

### Pyroptosis

Pyroptosis is an inflammatory programmed (i.e. regulated) lytic cell death characterized by the excessive release of proinflammatory cytokines leading to inflammation [143]. Gasdermins are members of a family of pore-forming effector proteins involved in pyroptotic cell death [19]. These proteins (6 members in humans: GSDMA, GSDMB, GSDMC, GSDMD, GSDME and PJVK), except PJVK (Pejvakin, DFNB59), lead to membrane permeabilization and ultimately lytic cell death, upon activation by certain stimuli [11, 19]. Gasdermins contain a cytotoxic N-terminal (NT) domain with an intrinsic pore-forming activity, and a C-terminal domain (CT) which represses pore-forming activity of NT domain in the absence of an activating signal (such as the cleavage by an inflammatory caspase such as caspase-1, -4, -5, -8 or -11) [19, 37, 52, 70, 112]. These two protein domains of gasdermins are connected by a central flexible linker which is cleaved by certain caspases upon induction by certain pathogen-derived or host-derived danger signals (PAMPs and DAMPs, respectively). Following the release of intramolecular inhibition on the NT domain due to proteolytic cleavage of the linker region by caspases, NT pore-forming domain of gasdermin localizes into cell membranes (also to organelle membranes such as mitochondrial outer membrane) to form large oligomeric pores, which results in the disruption of ion homeostasis and in the ultimate induction of cell death [3, 11, 76, 109]. From these pores, certain pro-inflammatory proteins such as IL-18 and IL-1β as well as other small DAMPs (damage-associated molecular patterns) can be released to the extracellular space. In later stages of lytic cell death, NINJ1 (Ninjurin)-mediated plasma membrane rupture enables the release of larger proteins such as LDH and HMGB1 [11, 68, 133].

NLR family pyrin domain containing 3 (NLRP3) is a protein responsible for the activation of the NLRP3 inflammasome, the most well-studied inflammasome complex, resulting in the production of inflammatory cytokines and the subsequent pyroptosis, upon sensing of pathogen-derived or host-derived danger signals [121]. Different caspases are functional in the specific cleavage and activation of certain gasdermins. These inflammatory caspases are activated by various inflammasomes such as NLRP3 inflammasome upon induction by different stimuli [24, 35, 37, 88, 106, 108]. For instance, GSDMD can be cleaved at a certain amino acid position by inflammatory caspases such as caspase-1, -4, -5, and -11 or by caspase-8 depending on cell type, context and stimuli. Similarly, caspase-3 and -8 have been found to be functional in the cleavage of GSDME and GSDMC, respectively, in particular scenarios [55, 101, 131]. It should also be highlighted here that the expression of the NT pore-forming domain of GSDMD or of some other gasdermin family members is sufficient to induce pyroptosis without the need of caspase activation [11, 70, 112].

Since primary ovarian insufficiency (POI) and polycystic ovary syndrome (PCOS) are the most common granulosa cell-related disorders in women, here I focused on pyroptotic cell death of granulosa cells in the context of these two endocrine disorders, as detailed below.

### Premature Ovarian Insufficiency (POI)

Premature ovarian insufficiency (POI), also known as premature ovarian failure (POF), is clinically defined as the exhaustion of ovarian reserve in women younger than 40 (i.e. the occurrence of menopause before the age of 40 years), and is a main cause of infertility in women of childbearing age [44]. POI is characterized by menstrual disturbance (amenorrhea (for at least four months) or oligomenorrhea) accompanied by increased gonadotropin levels (e.g. FSH > 25 IU/L) and decreased estradiol (E2) levels [86]. Around 1% of women under 40 suffer from POI, and its clinical etiology manifests with high heterogeneity. Although previous research have suggested that the causes of POI include mutations (certain variants), immune factors, and iatrogenic factors (chemotherapy such as with cisplatin, radiotherapy and ovarian surgery), the pathogenesis of POI is still largely unclear; therefore, no successful standard treatment for this disorder is currently available to the patients [61, 65]. Together with diminished ovarian reserve (DOR), these conditions are likely to lie along a continuum of degrees of decrease in ovarian reserve. Misregulated or abnormal activation of primordial follicles may lead to the exhaustion of the non-renewable pool of primordial follicles, resulting in POI or early menopause [103].

Abnormal inflammation also changes the normal dynamics of ovarian follicles, resulting in poor oocyte quality, anovulation, and related infertility [17, 26] POI is considered to be a pathological chronic inflammatory state that induces the abnormal expression of inflammatory factors of immune cells, granulocytes, and other sources, thus interfering with the normal course of follicular development [58]. NLRP3 inflammasome, caspase-1, and IL-1β expression are increased in GCs of patients with early onset ovarian insufficiency, and the fertility of female mice can be improved by inhibiting the expression of the NLRP3 inflammasome [60]. Besides, NLRP3 inflammasome expression gradually increases with the aging of mouse ovaries, pointing to inflammaging of ovaries [60]. Moreover, apoptosis levels of GCs are substantially higher in POI models, showing that GC death might contribute to the development of POI [92]. Besides apoptosis, pyroptotic death of granulosa cells has also been observed in diverse POI models [27, 28, 75, 137]. For instance, in GCs of POI mice, cyclophosphamide (exposure to which induces POI) promotes an increase in protein levels of NLRP3, ASC, (cleaved) caspase-1, IL-1β and GSDMD, markers of a pyroptotic event [27, 28, 75, 137]. In general, NLRP3 inflammasome-induced pyroptosis is activated during ovarian ageing, resulting in granulosa cell death, follicle depletion and ultimately ovarian dysfunction [60, 79, 137].

Based on the etiology of the disease, the widely used POI animal models can be currently classified as chemotherapy-induced POI models, autoimmune POI models, POI models of mental stress, and galactose-POI models, each with their specific advantages and disadvantages [34]. Among these models, chemotherapy-induced POI animal models are the most commonly used animal models in research [33]. Chemotherapeutic agents, such as cyclophosphamide, tripterygium glycosides, busulfan, cisplatin and doxorubicin (or their combinations), are commonly used to establish animal models of chemotherapy-induced POI due to their irreversible cytotoxicity towards ovaries, including damage to oocytes and follicular depletion [81]. The development of animal models of cyclophosphamide-induced POI is simple, requiring only a single dose of cyclophosphamide [34]. Similarly, a single intraperitoneal injection of combination of cyclophosphamide and busulfan is also used to establish a chemotherapy-induced POI model in mice, as it takes a short time to establish the model with high success rate [123, 136]. Immunization of mice with ZP3 (zona pellucida) glycoprotein is classically used to establish autoimmune POI mice models [34, 46]. This polypeptide induces the production of ZP3 polypeptide antibodies, which bind to ovarian ZP3 to cause an immune response and intervene with the exchange of information between oocytes and surrounding granulosa cells, resulting in ovarian atrophy, anovulation, and other manifestations including human POI [29, 34, 99, 110].

### Polycystic Ovary Syndrome (PCOS)

Polycystic ovary syndrome (PCOS) is a polygenic and polyfactorial reproductive endocrinopathy influenced by a combination of genetic and environmental factors, and it afflicts around 5–10% of women of reproductive age. This disease manifests as a complex interplay of endocrine and metabolic disorders (leading to infertility and recurrent miscarriages, insulin resistance, glucose intolerance, type 2 diabetes mellitus, dyslipidemia, obesity, hirsutism (growth of excessive male-pattern hair in women following puberty), acne and cardiovascular problems, and also psychological disorders such as anxiety and depression) characterized by the thickening of the ovarian capsule and stroma due to increased collagen deposition and fibrous tissue, hyperandrogenism (androgen excess) and a decrease in the production of progesterone and estrogens, anovulation (the absence of ovulation) or oligovulation, disordered gonadotropin secretion, ovarian dysfunction, follicular arrest and polycystic ovary morphology [6, 30, 40, 41, 74, 94, 113]. It is the most common endocrine disorder in women of reproductive age, and the most common cause of anovulatory infertility (accounting for nearly 80% of anovulatory infertility patients) [18]. In women with PCOS, despite the presence of high number of follicles, the amount of high-quality matured oocytes is highly limited, which hugely prevents their intrinsic ability of meiotic maturation and fertilization [32]. This is mostly due to abnormal development of follicles and growth arrest during the early antral phase [39].

Although the cause of PCOS remains largely unknown, previous research suggest that abnormal GC activity is closely associated with the pathogenesis of PCOS, and that apoptosis of GCs are increased in PCOS which induces the premature follicular atresia, i.e. the degeneration and resorption of several follicles and their ovules prior to the maturation and release of one ovule from a healthy follicle [96, 104]. GC death is correlated with unfavorable oocyte quality, ovary dysfunction, and low fertilization rates in PCOS [95, 154]. PCOS patients have also higher levels of low-grade chronic inflammation; for instance, inflammatory cytokines such as IL‐18 are highly expressed in these patients [130, 138]. Although apoptotic cell death does not generally induce inflammation, pyroptotic cell death is highly pro-inflammatory. Indeed, NLRP3 inflammasome activation and subsequent pyroptosis of GCs have a critical role in the pathophysiology of PCOS. Active NLRP3 inflammasome contributes to the development of PCOS, particularly in overweight patients [22]. PCOS process has been recently shown to be accompanied by the pyroptosis of GCs resulting from caspase-1 inflammasome activation [20]. NLRP3 and caspase-1 protein expression (both pro-caspase-1 and cleaved caspase-1) are significantly higher in GCs from patients with PCOS than in GCs from non-PCOS patients [129]. Pyroptosis of GCs is increased in PCOS women [135]. Although the viability of GCs from PCOS mice increases following the inhibition of pyroptosis, blockade of other cell death mechanisms (such as ferroptosis) does not influence GC survival, indicating the relative importance of pyroptotic death of GCs in the context of PCOS compared to other cell death mechanisms [20]. Cleavage of caspase 1 and GSDMD is enhanced in GCs from PCOS mice, and these cells release higher levels of IL-1β and IL-18 compared to GCs from control mice, all pointing to the increased pyroptotic cell death in the case of PCOS. In support, inhibition of caspase-1 activation increases the viability of GCs in PCOS mice, thus rescues the pathogenesis of PCOS [20]. Furthermore, hyperandrogenemia, one of the main pathophysiological changes that take place in patients with PCOS, results in GC pyroptosis in PCOS patients via the activation of NLRP3 inflammasome, in parallel to previous reports [150].

Naturally occurring nonhuman animal models of PCOS are currently unknown. Since hyperandrogenism is a major feature of PCOS, several androgens are used to induce PCOS‐like conditions (symptoms that resemble human PCOS, such as anovulation, cyst-like follicles, elevated LH levels, increased adiposity, and insulin insensitivity) in rodents, including testosterone, 5α‐dihydrotestosterone (DHT), and dehydroepiandrosterone (DHEA) [90, 102, 115]. DHT treatment is the most commonly used approach to establish a PCOS-like phenotype in rodents based on the fact that DHT is a non-aromatizable androgen and can not be converted into estradiol, unlike testosterone; thus, the effect can be fully attributed to DHT [126]. These androgenized animal models are typically established via prenatal or postnatal exposure to these exogenous androgens [42]. Prenatally androgenized animal models are usually developed via exposure to exogenous testosterone or dihydrotestosterone (DHT) during late gestation. Postnatal exposure to androgens is typically performed using DHT or DHEA at the prepubertal or pubertal stages. These exposures ultimately result in hyperandrogenism and PCOS-like phenotypes of the offspring in adulthood [42]. Alterations in the levels of kisspeptin and related molecules have been reported in the hypothalamus of these animal models of PCOS [90]. Besides androgens; estrogens, antiprogestin (progesterone receptor antagonists such as RU486 (mifepristone)), or aromatase inhibitors (for instance, letrozole) are used to generate rodent models of PCOS by subcutaneous injection or implantation. Each of these models have differences in terms of estrous cyclicity, ovarian morphology, gonadotropin and sex steroid profiles, neuropeptide levels in the hypothalamus, and certain metabolic features and adiposity [90]. For instance, prenatal DHT‐treated rats and mice have irregular estrous cycles and PCOS‐like ovarian morphology, increased LH levels with an upregulation of kisspeptin in the hypothalamus [90]. In addition to hormonally-induced mouse models of PCOS; transgenic (estrogen receptor knockout; aromatase knockout), diet-induced or chemically-induced mouse models of PCOS (for instance, by D-galactose, monosodium L-glutamate, bisphenol A or tributyltin) have also been described [31, 42, 90, 114, 126]. Prenatally androgenized rodents exposed to testosterone and letrozole-exposed rodents have been observed to most closely resemble the classic PCOS phenotype, whereas prenatally androgenized mice exposed to DHT better mimic the atypical PCOS patients [42].

## Factors that Induce Pyroptotic Cell Death in Ovarian Granulosa Cells

### Microplastics

Microplastics (MPs) are defined as plastics smaller than 5 mm in diameter. Polystyrene microplastics (PS MPs) induce the pyroptosis of ovarian granulosa cells via the NLRP3 / caspase-1 signaling pathway in Wistar rats [54]. At certain concentrations, polystyrene microplastics lead to increased protein levels of NLRP3, cleaved caspase-1, and cleaved GSDMD in addition to increased levels of IL-18 and IL-1β in rat granulosa cells, all pointing to the increased levels of pyroptotic cell death. Increases in the levels of these proteins are generally microplastic concentration-dependent. In addition to pyroptosis, apoptotic cell death events are also increased in response to polystyrene microplastics in rat granulosa cells, resulting in lower levels of growing follicles and ovarian reserve due to both programmed cell death pathways [54, 144]. PS MPs-induced NLRP3 inflammasome activation prior to pyroptotic cell death might be due to oxidative stress-induced phosphorylation of NF-κB which then activates NLRP3 inflammasome, based on the findings in this study that PS MPs lead to oxidative stress and disrupt antioxidant capacity in the ovaries of rats, and the previous reports showing that NF-κB is phosphorylated in response to oxidative stress [142], and that phosphorylated-NF-κB activates the NLRP3 inflammasome [147].

### Di-(2-Ethylhexyl) Phthalate (DEHP)

Di-(2-ethylhexyl) phthalate (DEHP) is an endocrine-disrupting chemical present in various consumer products including cosmetics, cleaning products, medical devices, textiles, and some other polyvinyl chloride (PVC) products, and this endocrine disruptor has also been observed to be present in the follicular fluid of women [49, 124]. DEHP contributes to ovarian dysfunction by inducing GSDMD-mediated pyroptotic cell death via the SLC39A5 / NF-κB / NLRP3 axis in GCs [119]. Mechanistically, DEHP leads to the overexpression of SLC39A5 which activates NF-κB pathway (higher protein levels of phosphorylated p65 (PP65)/P65 and TNF-α), followed by an increase in the transcript and protein expression levels of NLRP3, an increase in cleaved N-terminal GSDMD (which is able to form gasdermin pores in plasma membrane) protein levels, and also in increases in ratios of mature IL-1β / pro-IL-1β and caspase 1-p12 / pro-caspase 1 in GCs (in KGN cells and primary mouse GCs in vitro), resulting in diminished GC proliferation and impaired ovarian function, with more follicles undergoing atresia following exposure to DEHP. These pyroptotic factors are downregulated following the inhibition of NF-κB, pointing to the involvement of this axis in DEHP-mediated pyroptosis in GCs. DEHP-treated mice have also smaller reproductive organs (uterus and ovaries), fewer healthy follicles, and diminished ovarian reserve, showing that DEHP might negatively influence fertility in mice through increased pyroptotic death of GCs [119].

### Hyperandrogen (High Levels of Androgens)

Hyperandrogen induces chronic low-grade inflammation by inflammasome activation in PCOS mice, and leads to the pyroptotic death of ovarian GCs through the activation of the NLRP3 inflammasome [130]. DHT (dihydrotestosterone) treatment for 48 h at a certain concentration significantly increases NLRP3, ASC, activated caspase-1 (p10), GSDMD, GSDMD CT (cleaved GSDMD), IL-1β and IL-18 protein levels in GCs. GCs treated with 0.5 or 2 μM DHT show a rounding up of the cell body, and those treated with 5 μM DHT exhibit the swelling of the cell body and plasma membrane disruption. Besides, DHT mediates the release of LDH from GCs and decreases GC viability in a dose-dependent manner. Hyperandrogen-mediated NLRP3 activation promotes the pyroptosis of GCs, further exacerbating follicular dysfunction by dysregulating the expression of folliculogenesis- and steroidogenesis-related genes in GCs, and also drives ovarian fibrosis [130]. Furthermore, authors suggested that TLR4 pathway may mediate hyperandrogenism-induced chronic low-grade inflammation in PCOS mice, possibly by leading to the activation of NLRP3 inflammasome via the downstream signaling molecule MyD88 and activation of NF-κB [69, 130]. Increased ROS levels might also contribute to inflammasome formation in DHT-treated GCs [130].

Testosterone treatment (10 μM) also increases the mRNA and protein expression of IL-1β and NLRP3, and protein expression of IL-18 in human KGN cells, which also results in certain pyroptotic features including cell swelling and cell body rounding up, and formation of multiple bubble-like protrusions on the plasma membrane, and increased dose-dependent release of LDH [135]. Furthermore, testosterone upregulates the mRNA expression of ASC and caspase-1, and protein expression of caspase-1 and the ratio of GSDMD-NT / GSDMD, pointing that testosterone triggers pyroptosis in KGN cells in vitro [135]. These studies demonstrate that excess androgen might lead to female reproductive disorders such as PCOS by promoting pyroptotic death of GCs, in addition to some other potential mechanisms. Again, here, NF-κB whose expression is increased due to testosterone, and hyperandrogenism-induced endoplasmic reticulum (ER) stress can induce the formation of NLRP3 inflammasomes, ultimately leading to pyroptotic cell death in GCs [25].

### Non-Esterified Fatty Acids (NEFA)

High producing dairy cows experience a negative energy balance shortly after birth, which is associated with impaired reproductive performance, and which is also accompanied by high plasma concentrations of non-esterified fatty acids (NEFA) up to around 3 weeks post partum [66]. NEFA treatment increases the protein expression of NLRP3 and caspase-1 and the release of IL-1β in a dose-dependent manner in cow GCs, pointing that NEFA may contribute to pyroptotic death of cow GCs, leading to poor reproductive capacity [132]. Besides, NEFA stimulation induces oxidative stress, resulting in increased protein level of TLR4 and in the phosphorylation of NF-κB, and also increases the production of IL-6 and nitric oxide (NO), indicating that NEFA may induce inflammation in these cells. However, these NEFA-mediated effects can be partially reversed when the GCs are pre-treated with N-acetylcysteine (NAC), an antioxidant and radical scavenger. Pre-treatment with NAC limits NEFA-induced increases in NLRP3 and caspase-1 protein levels, and also suppresses the release of IL-1β in GCs. This shows that NEFA can induce pyroptosis and inflammation through NLRP3 inflammasome and oxidative stress-NF-κB pathway, respectively; and NAC can alleviate these conditions to a particular extent [132]. This study also supports other findings detailed above that oxidative stress induced by various factors and TLR4/NF-κB axis is involved in the pyroptotic death of GCs.

### High-Fat Diet and Letrozole

Letrozole is an aromatase inhibitor used in the treatment of breast cancer, a hormone-dependent cancer such as ovarian cancer [13, 15, 16]. Letrozole functions by inhibiting the action of aromatase, the enzyme which converts androgens into estrogens by a process called aromatization [12]. Letrozole treatment increases NLRP3 protein levels in GCs of mice compared to control and high-fat diet-fed mice [129]. GCs of both letrozole treated- and high-fat diet-fed mice show increased caspase-1 protein levels compared to GCs of control mice, or compared to GCs of only letrozole treated- or of only high-fat diet-fed mice. The serum concentrations of IL-18 and IL-1β in mice treated with high-fat diet + letrozole are also significantly higher compared to that in only high-fat diet-fed mice. Besides, both letrozole treated- and high-fat diet-fed mice have increased number of cystic follicles and decreased number of corpora lutea compared to control mice [129]. These observations suggest that high-fat diet and treatment with letrozole might result in increased pyroptotic events in GCs of mice, leading to unfavorable reproductive health in female mice. Authors also suggested that NLRP3 may influence follicle development by regulating the expression of proteins involved in the glycosylation of many hormones and growth factors that are indispensable for oogenesis and folliculogenesis [129].

### Local Glucose Elevation

Glucose activates NLRP3 inflammasome and pyroptosis in a dose- and time-dependent manner in GCs from patients [140]. It increases NLRP3, ASC, caspase 1, IL-1β mRNA levels, NLRP3, ASC and cleaved IL-1β protein levels, caspase 1 activity, and GSDMD mRNA and NT-GSDMD protein levels, generally in a glucose concentration-dependent manner (from 5 to 35 mM), in GCs, resulting in decreased cell viability at higher concentrations of glucose. Besides, the inhibition of NLRP3 restricts pyroptotic cell death induced by high glucose in GCs, as shown by, for instance, decreased NT-GSDMD protein abundance and lower LDH release [140].

TXNIP facilitates the oligomerization of NLRP3 and stabilizes the inflammasome complex [156]. High glucose treatment stimulates TXNIP mRNA and protein expression in GCs, and knockdown of TXNIP results in reversing the upregulation of NLRP3 inflammasome and certain pyroptosis biomarkers induced by high glucose treatment, pointing that TXNIP participates in high glucose-induced activation of NLRP3 inflammasome and pyroptosis in GCs [140]. Moreover, the activation of NLRP3 inflammasome and pyroptosis impaires estradiol (E2) synthesis in GCs, and decreased E2 synthesis is also observed following high glucose treatment in these cells. In contrast, inhibition of pyroptosis reverses high glucose-induced downregulation of E2 levels in GCs [140]. This points that increased NLRP3 inflammasome activation and pyroptotic cell death induced by higher local glucose may be a potential reason for impaired steroidogenesis and decreased E2 synthesis in human GCs.

Clinically, overweight women have elevated follicular glucose levels which significantly induce NLRP3 inflammasome formation and pyroptotic events in GCs, as shown by, for instance, higher NLRP3, ASC, and cleaved-caspase-1 protein levels, increased caspase-1 activity, higher IL-1β levels in follicular fluid, increased levels of GSDMD mRNA and NT-GSDMD protein, and characteristic pore formation in GC plasma membranes [140]. This shows that high glucose might be a potent inducer of pyroptotic death of GCs, and a specific mediator of obesity-associated reproductive issues in women.

## Factors that Limit Pyroptotic Cell Death in Ovarian Granulosa Cells

### Plumbagin

Plumbagin (5-hydroxy-2-methyl-1,4-naphthoquinone) is a compound isolated from the root of the plant *Plumbago zeylanica L.,* also known as chitrak [5, 105]. Administration of plumbagin prevents the pyroptosis of GCs and the onset of PCOS, by suppressing WTAP-mediated N6-methylation of ASC mRNA [20]. Normally, the upregulation of WTAP (a key regulator of RNA N6-methylase complex) results in the overactivation of the inflammasome in GCs, since WTAP stabilizes ASC (a NLRP3 inflammasome component) mRNA by mediating its N6-methylation in GCs from the PCOS model. However, plumbagin reverses this inflammasome overactivation by destabilizing ASC mRNA, decreasing its mRNA and protein levels, in GCs isolated from the ovary of mice. It thus decreases the cleavage of both caspase-1 (resulting in the suppression of caspase-1 activation) and GSDMD (resulting in the supression of pyroptosis), and the release of IL-1β, IL-18 and LDH in GCs, which are all normally increased in the case of PCOS. In summary, plumbagin lowers pyroptotic death of granulosa cells, which is normally heightened in the case of PCOS [20].

### Quercetin

Cyclophosphamide, a chemotherapeutic drug, damages the ovaries in a dose-dependent manner [91, 92]. Recent studies have demonstrated that cyclophosphamide-induced ovary damage is associated with the facilitation of GC apoptosis [145]. Quercetin, a natural flavonoid widely found in many fruits and plants, including tea, onions, apples, and kale, protects the ovarian reserve from damage due to cyclophosphamide-induced POI by downregulating pyroptosis in mice [27]. More specifically, quercetin reduces the protein levels of NLRP3, GSDMD, caspase-1 and IL-1β in the GCs of cyclophosphamide-induced POI mice, possibly resulting in the inhibition of pyroptosis and in the limitation of cyclophosphamide-induced ovarian damage [27]. Again, in this context, mitochondrial ROS-mediated activation of inflammasomes might be involved in the pyroptosis of GCs, since the expression of oxidative stress products are increased, whereas that of antioxidant products are decreased in this induced POI model, which are reversed after quercetin treatment [27, 51, 141].

### HDAC1 (Histone Deacetylase 1)

HDAC1 suppresses the pyroptosis of GCs in PCOS through deacetylation of H3K9ac on the lncRNA H19 promoter to regulate the H19 / miR-29a-3p / NLRP3 axis, as shown in in vitro PCOS cell models (dihydrotestosterone (DHT)-induced HGL5 cells) [26]. HDAC1 expression is lower in patients with PCOS compared to those with non-PCOS at both mRNA and protein level; and its overexpression inhibits PCOS-induced pyroptosis due to decreased protein levels of NLRP3, GSDMD-NT, and cleaved caspase-1, IL- 1β and IL-18, and thus alleviates PCOS phenotype in mouse models (established using dehydroepiandrosterone (DHEA)). Mechanistically, in in vitro PCOS models, HDAC1 inhibits lncRNA H19 transcription through H3K9 deacetylation on H19 promoter, and reduces the binding of H19 to miR-29a-3p to inhibit NLRP3 expression, thus suppressing GC pyroptosis [26]. HDAC1 is also negatively correlated with the levels of certain inflammation markers (IL-6, IL-1β, IL-18, and TNF-α) in the serum of PCOS patients, among which IL-1β and IL-18 are mostly released from cells during pyroptosis, suggesting the association between HDAC1 and pyroptotic cell death in PCOS patients, and anti-pyroptotic function of HDAC1 via H19/miR-29a-3p/NLRP3 axis [23]. This study shows that epigenetic regulators might also be responsible in the regulation of pyroptotic GC death in PCOS.

### Leonurine Hydrochloride

Leonurine (4-guanidino-n-butyl syringate), an alkaloid from *Leonurus heterophyllus*, inhibits the over-activation of NLRP3 inflammasome [77]. Intraperitoneal administration of leonurine hydrochloride prevents cyclophosphamide-induced POI by inhibiting NLRP3 / GSDMD-mediated pyroptosis of GCs and improves cyclophosphamide-induced infertility in mice [28]. Leonurine hydrochloride treatment normalizes the ovarian protein expression of NLRP3, ASC, cleaved caspase-1, and GSDMD, which is increased by cyclophosphamide, and also significantly lowers the levels of serum IL-18 and IL-1β. Furthermore, leonurine hydrochloride treatment increases the weight of reproductive organs, the numbers of primordial follicles, primary follicles, and secondary follicles, reduces the number of atretic follicles, and increases the numbers of live fetuses and implantations (fertility-related indicators) [28]. These observations suggest that leonurine hydrochloride might lead to improved fertility in POI by limiting pyroptosis in GCs, at least in mouse models. Leonurine has been shown to have strong binding ability to PI3K and to suppress IL-1β-induced activation of the PI3K/Akt/NF-κB signaling pathway or to lead to NF-κB inactivation in general [56, 73, 78]. Considering the ability of active NF-κB to activate the NLRP3 inflammasome [147], anti-pyroptotic functionality of leonurine might be due to decreased NF-κB-mediated activation of NLRP3 inflammasome, ultimately resulting in a smaller number of pyroptotic events in GCs.

### Bushenhuoluo Decoction (BSHLD)

Bushenhuoluo Decotion (BSHLD) is a traditional empirical formula (of Traditional Chinese medicine (TCM)) which consists of 14 traditional Chinese herbs including Shudihuang, Danggui, Chuanxiong, Baishao, Nvzhenzi, Hanliancao, Tusizi, Chongweizi, Fupenzi, Yinyanghuo, Taoren, Honghua, Lulutong and Huainiuxi [57]. Bushenhuoluo Decoction treatment inhibits NLRP3-mediated GC pyroptosis in ovarian tissues of PCOS rats, thus improving ovarian function, by regulating exosomal miR-30a-5p / SOCS3 (suppressor of cytokine signaling 3) / mTOR / NLRP3 signaling [57]. BSHLD reduces miR-30a-5p expression in serum, serum exosomes and ovarian tissues in PCOS rats, and decreased exosomal miR-30a-5p attenuates LPS-triggered pyroptosis in GCs. Mechanistically, miR-30a-5p directly targets SOCS3 to inactivate mTOR / P70S6K signaling. BSHLD administration significantly reverses IL-1β and IL-18 production in ovarian tissues of PCOS rats, in parallel to decreases in NLRP3, ASC, and caspase 1 mRNA levels and in NLRP3 protein levels, pointing the potential of Chinese herbal medicine BSHLD as an alternative for the treatment of PCOS, by limiting pyroptotic cell death of GCs. In contrast, mTOR agonist rapamycin reverses miR-30a-5p inhibitor (miR-30a-5p antagomir)-mediated decreases in pyroptosis, leads to increased NLRP3 levels and pyroptotic events in GCs. In other words, the inhibitory effect of miR-30a-5p deficiency on GC pyroptosis is attenuated by rapamycin, leading to increased pyroptotic cell death of GCs [57]. This shows that BSHLD and rapamycin might function in reverse directions in the regulation of GC pyroptosis. Here, it should also be noted that miR-30a-5p could drive NF-kB/NLRP3 signaling pathway, and that SOCS family members are capable to regulate various signaling pathways including NF-kB pathway, in some other contexts [93, 134].

### Bushen Cuyun Recipe

Bushen Cuyun Recipe (BCR) is an ancient Chinese herbal decoction and a product marketed in China (with tradename of Yueling Yin) with proposed positive effects on female infertility [64]. It comprises of 10 different herbs [64]. Bushen Cuyun Recipe protects GCs against pyroptosis in the case of diminished ovarian reserve induced by cyclophosphamide in rats, by reversing the elevated protein levels of GSDMD and caspase-1 in GCs, suggesting that the inhibition of pyroptosis by BCR could ameliorate ovarian function in the case of diminished ovarian reserve. This treatment relieves the reduction in the numbers of growing follicles and corpus lutea from last ovulation, potentially leading to improved fertility in rats [64]. Authors showed in another study by performing molecular docking analyses that the main active components of BCR (kaempferol, quercetin, and hesperetin) can stably bind to GSDMD, resulting in decreased levels of available GSDMD [63]. Therefore, it can be speculated that BCR or its certain components more directly limit pyroptotic death of GCs by possibly binding to its main executioner proteins (i.e. gasdermins) and preventing its oligomerization to be able to form membrane pores.

### α-Ketoglutarate

α-Ketoglutarate is a key intermediate in the tricarboxylic acid (TCA) cycle, also known as Krebs cycle, a series of chemical reactions used to generate energy by the oxidation of acetylcoenzyme A (CoA) derived from carbohydrates, fatty acids and proteins in aerobic organisms. α-Ketoglutarate treatment reduces the expression of inflammatory factors in the ovaries and reduces systemic inflammatory cytokines, thus inhibiting the level of intracellular inflammation [62, 78, 111, 151]. Long-term use of α-ketoglutarate delays the decline of fertility in mice and improves the number and quality of follicles and oocytes [151]. α-Ketoglutarate improves ovarian reserve function in POI by inhibiting NLRP3-mediated pyroptosis of GCs [75]. It decreases mRNA levels of NLRP3, caspase-1, GSDMD and IL-18, and protein levels of NLRP3, cleaved-caspase 1, GSDMD, IL-1B and IL-18, which are normally increased in cyclophosphamide-induced POI models compared to controls, in rat GCs [75]. In vitro, in KGN cells (ovarian granulosa cell tumor cell line) treated with LPS and nigericin (microbial toxin derived from the gram-positive bacteria *Streptomyces hygroscopicus,* used to trigger the NLRP3 inflammasome) to mimic pyroptosis, α-ketoglutarate (2 mM) limits pyroptotic cell death (as quantified by decreased LDH release from cells), decreases the released IL-18 and IL-1β protein levels, and lowers inflammatory and pyroptosis factors such as NLRP3, GSDMD, caspase-1, IL-18 and IL-1β at both mRNA and protein levels, showing that α-ketoglutarate could delay or even reverse cyclophosphamide-induced POI by inhibiting NLRP3-mediated pyroptosis in granulosa cells [75]. Authors also reported that α-ketoglutarate intervention elevates lactate levels in POI rats, thereby restoring the expression of certain glycolytic enzymes. Since the disruption of glycolytic flux is a signal for inflammasome signaling and pyroptosis mostly due to decreased NADH levels and induction of mitochondrial ROS production [107], the restoration of glycolysis or downstream TCA cycle metabolism with the supplemantation of specific metabolites such as α-ketoglutarate might limit inflammasome activation and downstream pyroptotic cell death in GCs.

### Human Bone Marrow Derived-Mesenchymal Stem Cells (hBMSCs)

Autoimmune-related disorders are responsible for approximately 4%-30% of POI cases [43]. In vitro, co-culture of hBMSCs (human bone marrow derived-mesenchymal stem cells) with autoimmune-damaged GCs (modeled by IFN-γ-induced human GC line KGN) reduces the occurrence of pyroptosis in GCs in autoimmune POI [83]. In more detail, hBMSC cell co-culture reduces the level of LDH released (a pyroptotic cell death marker, as a marker of cell membrane breakdown) from KGN cells, and also decreases NLRP3 and caspase-1 mRNA levels, and caspase-1 protein levels, suggesting that hBMSCs might repair IFN-γ-induced autoimmune damage in KGN cells occurred due to pyroptotic cell death [83]. hBMSCs co-culture treatment does not change NLRP3 and GSDMD protein levels; however, it decreases caspase-1 protein levels in KGN cells. Therefore, it can be suggested that hBMSCs co-culture treatment limits pyroptotic cell death of GCs mostly by decreasing the processing and activation of GSDMD (free NT domain of GSDMD formed following caspase cleavage is able to form pores), rather than regulating its protein levels. In vivo, hBMSCs improve estrous cycle disorders in autoimmune POI mice, increases serum estradiol levels, decreases serum FSH levels, improves ovarian function and morphology, increases the number of primordial, primary and mature follicles, and decreases the number of atretic follicles [83]. Some of these in vivo effects in mice might be attributed, at least to a certain extent, to decreased pyroptotic events in GCs following hBMSC treatment.

Exosomes derived from bone marrow mesenchymal stem cells also suppress NLRP3/Caspase1/GSDMD pyroptosis axis in vitro (KGN cells exposed to IFN-gamma) and in vivo (BALB/c mouse model of autoimmune POI, induced by zona pellucida glycoprotein 3 (ZP3)), resulting in the restoration of ovarian function (normalization of the irregular oestrous cycles, rescue of the follicular loss, and increased the pregnancy rate and number of offspring) in autoimmune POI [137]. Exosomes derived from these mesenchymal stem cells downregulate the expression of the NLRP3, caspase 1 and GSDMD, decrease the level of LDH released, and also inhibit activation of the NF-κB pathway in ovarian granulosa cells, indicating that exosomal treatment promotes the function of ovarian granulosa cells by suppressing the NF-κB pathway [137]. Furthermore, the rising levels of IL-1β in the serum of the POI group (compared to controls) significantly decrease after this exosomal treatment in mice [137].

### He’s Yang Chao Recipe

He’s Yang Chao Recipe (HSYC) is a traditional Chinese medicine originated from ‘Cong Rong Tu Si Wan’, a classic and famous formula recorded in the traditional Chinese medicinal book Ji-Yin-Zong-Lu in the period of Ming Dynasty. This recipe primarily contains eight herbs [85]. Treatment with He’s Yang Chao recipe significantly reduces the mRNA and protein expression of NLRP3, caspase-1, GSDMD, IL-18, and IL-1B in GCs of cyclophosphamide-induced POI mouse models [85]. This points that He’s Yang Chao recipe protects GCs in mice with POI potentially by the contribution from the inhibition of NLRP3 inflammasome activation [85]. The mechanism by which He’s Yang Chao recipe limits pyroptotic death of GC in mice remains to be studied. Besides, specific molecules of this recipe which are particularly functional in the regulation of pyroptosis in GCs should be identified in future studies.

### Metformin

Cisplatin is a DNA-damaging chemotherapy drug used in the first-line standard treatment of ovarian cancer, in addition to the treatment of some other solid malignant tumors [14]. However, it can adversely affect ovarian health and fertility (for instance, by reducing ovarian primordial follicles, decreasing ovarian reserve and diminishing the number of sinusoidal follicles) in premenopausal cancer patients; thus, the ovarian endocrine and reproductive functions of female patients with cancer should be safeguarded against cisplatin-induced damage. Metformin is a biguanide antihyperglycemic agent and first-line pharmacotherapy used in the management of type II diabetes [127]. It is also used off-label for the treatment of insulin resistance in PCOS [4]. Besides, metformin extends average lifespan in certain model organisms [10]. Metformin mitigates cisplatin-induced ovarian damage by the inhibition of GC pyroptosis via ROS/TXNIP/NLRP3 signaling pathway [128]. The number of GC pyroptosis events significantly decreases in the cisplatin + metformin compared to only cisplatin group, in parallel to decreases in the protein levels of caspase-1, GSDMD, IL-1β, and in mRNA and protein levels of TXNIP and NLRP3 [129]. Treatment with a TXNIP inhibitor results in the loss of metformin-induced decreases in cisplatin-induced pyroptotic cell death in GCs. In summary, metformin can, to a certain extent, inhibit GC pyroptosis due to cisplatin, potentially by the inhibition of the TXNIP / NLRP3 / caspase-1 / GSDMD axis in mice [128]. Metformin also downregulates cellular levels of ROS which is known to induce NLPR3 inflammasome activation, and to augment S-palmitoylation of GSDMD which is required for it to be able to form pores [1, 38].

Metformin also inhibits human KGN cell pyroptosis via the miR-670-3p/NOX2/ROS pathway [155]. It decreases NLRP3 and ASC mRNA levels, and NLRP3, cleaved caspase-1, ASC and GSDMD-NT protein levels, released LDH levels and caspase-1 activity in LPS-treated human KGN cells. Some of these metformin-mediated decreases in pyroptotic factors are further decreased with additional treatment with N-acetyl-L-cysteine (NAC), a ROS scavenger. Mechanistically, metformin upregulates miR-670-3p which inhibits the NOX2-ROS axis by targeting NOX2 in KGN cells, leading to the repression of GC pyroptosis in vitro [155].

### Genipin and Tauro-ursodeoxycholic Acid (TUDCA)

Genipin (GP), an inflammation inhibitor which was initially isolated from the Chinese medicinal plant *Gardenia jasminoides* [97], suppresses testosterone-induced inflammation in human GCs (KGN cells) [135]. Genipin decreases mRNA levels of IL-1β, NLRP3, ASC and caspase-1, and protein levels of IL-18, IL-1β, NLRP3, caspase-1 and the ratio of GSDMD-NT/GSDMD, and also reduces released LDH levels, showing that the suppression of inflammation attenuates androgen-induced pyroptosis in GCs in vitro [135]. Similar reduction in the mRNA and protein levels of pyroptosis-related factors and in LDH release are observed following the treatment of KGN cells with TUDCA (a bile acid and an inhibitor of ER stress), resulting in the suppression of testosterone-induced inflammation in these cells [135]. This points that ER stress might mediate testosterone-induced pyroptosis and inflammation in KGN cells. ER stress is known to promote NLRP3 inflammasome activation through multiple mechanisms including the unfolded protein response (UPR), calcium or lipid metabolism, and ROS generation [25, 71, 89, 122]. The limiting effect of ER stress inhibition on pyroptotic death of GCs might be mainly due to decreased activation of NLRP3 inflammasome, possibly resulting in decreased caspase-1 activation and pro-IL-1β, pro-IL-18 and GSDM processing.

### Cysteine-rich 61 (CYR61)

Rats with premature ovarian failure have lower levels of Cysteine-rich 61 (CYR61; also known as CCN1) in their GCs [139]. This protein limits cyclophosphamide-induced inhibition of proliferation in rat GCs by suppressing NLRP3 / caspase 1-mediated pyroptotic cell death [139]. Overexpression of CYR61 decreases NLRP3, caspase 1, GSDMD and IL-1β protein levels, whereas its silencing results in increased expression of these proteins in cyclophosphamide-induced GCs [139]. CYR61 overexpression leads to higher viability of cyclophosphamide-treated rat GCs, whereas CYR61 silencing results in decreased viability of these cells [139]. These observations suggest that CYR61 is involved in the suppression of pyroptotic death of GCs induced by cyclophosphamide, leading to increased GC viability. CYR61 also inhibits cyclophosphamide-induced senescence of rat ovarian granulosa cells (as shown by downregulation of senescence markers p53 and p21, and less SA-β-gal positivity), and mechanistically this may be related to the regulation of caspase-1/NLRP3-induced pyroptosis. This can be supported by previous research demonstrating a potential association between cellular senescence and pyroptosis [47, 53, 67, 72, 152, 157].

### Cyproterone Acetate

Cyproterone acetate (CYA) is a progestin-based drug frequently used to reduce androgen in PCOS patients. Cyproterone acetate treatment decreases the expression of pyroptosis-related proteins in mice GCs. More specifically, cyproterone acetate leads to decreases in the protein levels of NLRP3, GSDMD, GSDMD-NT, caspase 1 and cleaved-caspase 1 in GCs from mice with hyperandrogen-induced PCOS, in addition to decreases in IL-18 and IL-1β concentration in serum [150]. Mechanistically, in vitro, cyproterone acetate ameliorates the pyroptosis of KGN cells by decreasing the activation of the IRE1 signaling pathway [150]. Activated IRE1 increases TXNIP mRNA stability, and in turn, elevated TXNIP protein activates the NLRP3 inflammasome [71, 100, 122]. This IRE1-TXNIP axis-regulated NLRP3 inflammasome activation has been also shown to be manipulated in the case of other pyroptosis-inducer or -inhibitor factors in granulosa cells as detailed above.

**Fig. 1:**
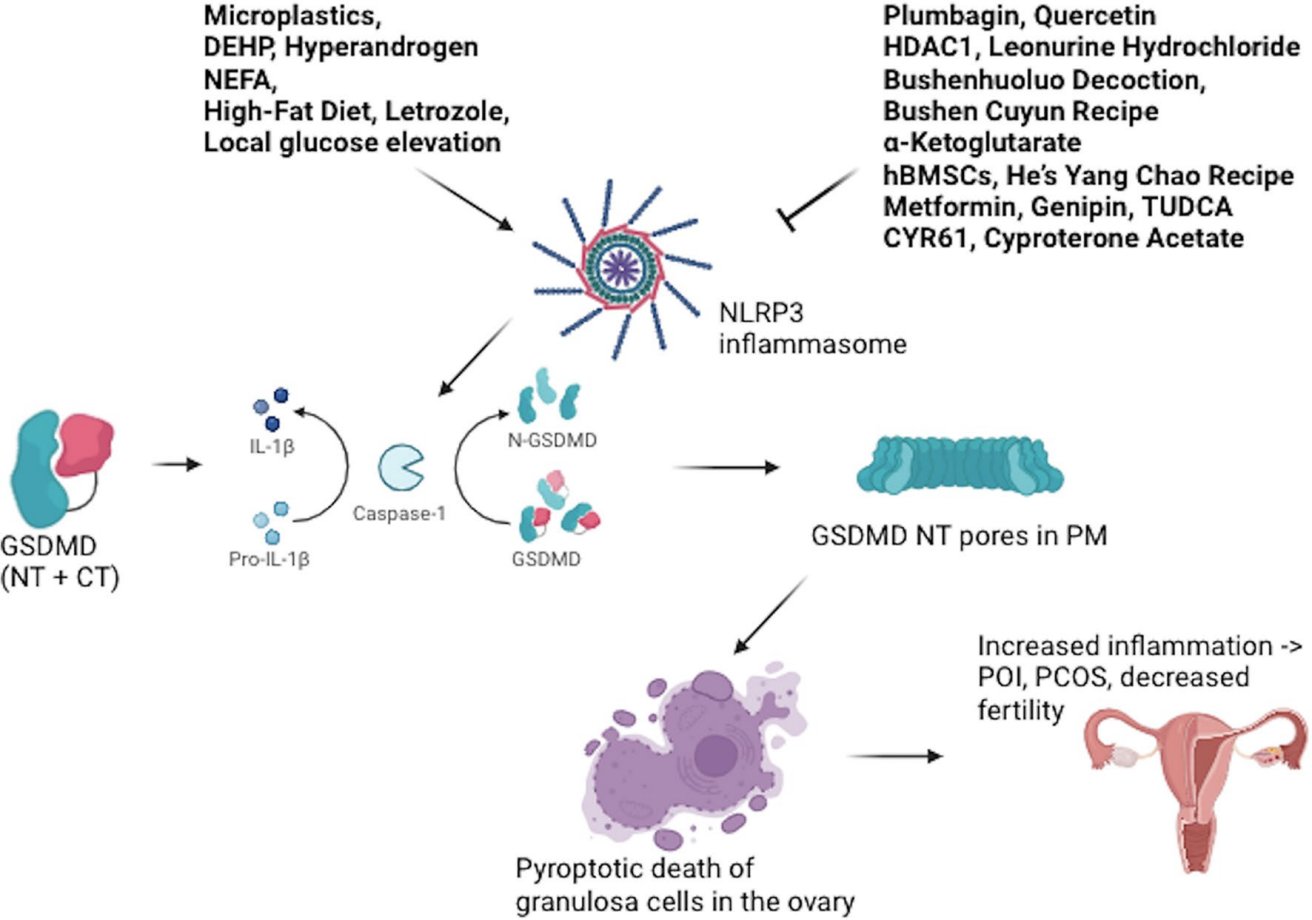
Pyroptosis in ovarian granulosa cells (GCs), pyroptosis-inducers and -inhibitors in these cells, in various models of Premature Ovarian Insufficiency (POI) and Polycystic Ovary Syndrome (PCOS). Both pyroptosis-inducer and -inhibitor factors mostly influence NLPR3-, caspase 1- and GSDMD-mediated pyroptosis in GCs in vitro and in vivo models of POI and PCOS. In the presence of a pyroptosis inducer (such as microplastics), caspase 1, following its activation by NLRP3 inflammasome, cleaves GSDMD between its N-terminal and C-terminal domains (NT and CT, respectively), and then free GSDMD-NT domains oligomerize to form pores in the plasma membrane leading to the pore-mediated release of certain pro-inflammatory molecules including IL-18 and LDH, and ultimately leading to lytic pro-inflammatory cell death

A summary of pyroptosis-inducer and -inhibitor factors in granulosa cells in diverse models systems of POI and PCOS is given in Fig. 1 and Table 1.

**Table 1:** Summary table showing pyroptosis inducing or limiting factors, associated molecular changes and pathways, studied models / cell types and certain reproductive changes observed

Factor	Induce / limit pyroptosis	Molecular changes / pathway / signaling axis	Animal / cell type / disease model	Reproductive changes	Reference
Polystyrene (PS) microplastics (MPs)	induce	increased protein levels of NLRP3, cleaved caspase-1, and cleaved GSDMD, p-NF-κB, and of IL-18 and IL-1β	Wistar rats	lower levels of growing follicles and ovarian reserve; thinning of the thickness of the granulosa layer of some secondary follicles	[54]
Di-(2-Ethylhexyl) phthalate (DEHP)	induce	via SLC39A5 / NF-κB / NLRP3 axis; increased NLRP3 mRNA and protein levels, cleaved N-terminal GSDMD protein levels, and increases in ratios of mature IL-1β/ pro-IL-1β and caspase 1-p12 / pro-caspase 1, increased protein levels of relative protein levels of phosphorylated p65 (PP65)/P65 and TNF-α	KGN cells and primary mouse GCs	ovarian dysfunction, diminished GC proliferation with more follicles undergoing atresia, smaller reproductive organs (uterus and ovaries), fewer healthy follicles, and diminished ovarian reserve, more irregularly shaped follicles	[119]
DHT (dihydrotestosterone), testosterone	induce	increase in NLRP3, ASC, activated caspase-1 (p10), GSDMD, GSDMD CT (cleaved GSDMD), IL-1β and IL-18 protein levels, increased TLR4 expression	C57BL/6 J mice	follicular dysfunction, dysregulation in the expression of folliculogenesis- and steroidogenesis-related genes, ovarian fibrosis, increased expression offibrotic factors in ovarian cells	[130]
Testosterone	induce	Increases in the mRNA and protein expression of IL-1β and NLRP3, and protein expression of IL-18, increased dose-dependent release of LDH, upregulation of mRNA expression of ASC and caspase-1, and increased protein expression of caspase-1 and increased ratio of GSDMD-NT/ GSDMD; ER stress sensor proteins	human KGN cells; dehydroepiandrosterone(DHEA)-treated mouse PCOS model, clinical specimens (from PCOS patients with hyperandrogenism)		[135]
Non-Esterified Fatty Acids (NEFA)	induce	increases in the protein expression of NLRP3 and caspase-1 and the release of IL-1β, induction of oxidative stress, the phosphorylation of NF-κB, and increases in the production of IL-6 and nitric oxide (NO), higherprotein expression of TLR4 and p-p65	cow GCs	poor reproductive capacity	[132]
High-Fat Diet and Letrozole	induce	increases in NLRP3 and caspase-1 protein levels, increased serum concentrations of IL-18 and IL-1β	PCOS-like mice model	increased number of cystic follicles and decreased number of corpora lutea	[129]
Local glucose elevation	induce	NLRP3 inflammasome activation, increases in NLRP3, ASC, caspase 1, IL-1β mRNA levels, NLRP3, ASC and cleaved IL-1β protein levels, caspase 1 activity, and GSDMD mRNA and NT-GSDMD protein levels, higher LDH release; increased TXNIP mRNA and protein expression	GCs isolated from patients either normal weight or overweight	impaired estradiol (E2) synthesis, impaired steroidogenesis	[139]
Plumbagin (5-hydroxy-2-methyl-1,4-naphthoquinone)	limit	suppression of WTAP-mediated N6-methylation of ASC mRNA, destabilization of ASC mRNA, decreases in ASC mRNA and protein levels, reversal of inflammasome overactivation, decrease caspase 1 activation, decreased GSDMD level, decreased IL-1β and IL-18 levels	primary GCs isolated from the ovary of mice	Normalisation of thickness and arrangement of the GC layer in mice ovaries, normalisation of hormone levels	[20]
Quercetin	limit	reduction in the protein levels of NLRP3, GSDMD, caspase-1 and IL-1β, improved mitochondrial redistribution, upregulation of peroxisome proliferator-activated receptor gamma coactivator 1-alpha (PGC1α),mitochondrial transcription factor A (TFAM), and SOD2 expression at the mRNA and protein levels	GCs of cyclophosphamide-induced POI mice	limitation of cyclophosphamide-induced ovarian damage, elevated serum anti-Müllerian hormone, estradiol, and progesterone levels, decreased serum follicle-stimulating hormone and luteinizing hormone levels, and alleviation of ovarian pathology, increased number of growing follicles at all levels, decrased number of atretic follicles, and numerous GCs in mature follicles neatly arranged in a radial pattern	[27]
HDAC1 (Histone Deacetylase 1)	limit	deacetylation of H3K9ac on the lncRNA H19 promoter to regulate the H19/ miR-29a-3p/NLRP3 axis; decreased protein levels of NLRP3, GSDMD-NT, and of cleaved caspase-1, IL- 1β and IL-18	in vitro PCOS cell models (dihydrotestosterone(DHT)-induced HGL5 cells); dehydroepiandrosterone (DHEA)-induced mice models of PCOS	alleviation of PCOS phenotype, improved estrus cycle and HOMA-IR (homeostasis model of insulin resistance), pathological changes in ovarian structures were repaired, reducedd evels of TT and LH, increased FSH level	[26]
Leonurine Hydrochloride (4-guanidino-n-butyl syringate)	limit	Inhibition of the over-activation of NLRP3 inflammasome, inhibition of NLRP3 / GSDMD-mediated pyroptosis, lower ovarian protein expression of NLRP3, ASC, cleaved caspase-1, and GSDMD, lower levels of serum IL-18 and IL-1β	Mice models of cyclophosphamide-induced POI	Prevention of cyclophosphamide-induced POI, improvements in cyclophosphamide-induced infertility in mice, increases in the weight of reproductive organs, the numbers of primordial follicles, primary follicles, and secondary follicles, reductions in the number of atretic follicles, and increases in the numbers of live fetuses and implantations; normalisation of hormone levels	[28]
Bushenhuoluo Decoction (BSHLD)	limit	exosomal miR-30a-5p / SOCS3 / mTOR / NLRP3 signaling, decreased IL-1β and IL-18 production, decreases in NLRP3, ASC, and caspase 1 mRNA levels and in NLRP3 protein levels	ovarian tissues of PCOS rats induced by dehydroepiandrosterone (DHEA) (Sprague–Dawley rats) (daily subcutaneousinjection with DHEA for 20consecutive days), GCs isolated from these rats	improved ovarian function, improved serum hormone abnormality, insulin sensitivity, and ovarian morphologicchanges, decreased number of primary follicles and cystic follicles, increased number of corpus luteum	[57]
Bushen Cuyun Recipe (BCR)	limit	reversal of the elevated protein levels of GSDMD and caspase-1	rat model of diminished ovarian reserve induced by cyclophosphamide (intraperitoneal injection ofcyclophosphamide90 mg/kg once)	improved ovarian function, increased numbers of growing follicles and corpus lutea from last ovulation, improved ovarian morphology, increased ovarianindex, improved estrous cycle, and improved serumAMH, decreased serum FSH, increased serum GnRH and E2	[64]
α-Ketoglutarate	limit	decreases in mRNA levels of NLRP3, caspase-1, GSDMD and IL-18, and protein levels of NLRP3, cleaved-caspase 1, GSDMD, IL-1B and IL-18, decreased LDH release, decreased levels of the released IL-18 and IL-1β protein levels	cyclophosphamide-induced POI rat models (Sprague Dawley rats), KGN cells (ovarian granulosa cell tumor cell line)	improved ovarian reserve function in POI, improved estrous cycle and serum hormone levels, improved ovarian morphology	[75]
Human Bone Marrow Derived-Mesenchymal Stem Cells (hBMSCs) / exosomes derived from these cells	limit	reduced level of LDH released, decreased levels of NLRP3 and caspase-1 mRNA levels, and caspase-1 protein levels	autoimmune-damaged GCs (modeled by IFN-γ-induced human GC line KGN), autoimmune POI mice model (BALB/c mouse model induced by zona pellucida glycoprotein 3)	improved estrous cycle disorders in autoimmune POI mice, increased serum estradiol levels, decreased serum FSH levels, improved ovarian function and morphology, increases in the number of primordial, primary and mature follicles, and decreased number of atretic follicles	[83, 137]
He’s Yang Chao Recipe (HSYC)	limit	reduced mRNA and protein expression of NLRP3, caspase-1, GSDMD, IL-18, and IL-1B	GCs of cyclophosphamide-induced POI mouse models (C57/BL6, intraperitoneal injection)	protection of GCs in mice with POI, lower ovarian damage, increased total antioxidantcapacity, less oxidative stress, improved mitochondrial distribution and membrane potential levels, increased AMH levels, decreased FSH and LH levels, increased number of primary follicles	[85]
Metformin	limit	decreases in the protein levels of caspase-1, GSDMD, IL-1β, and in mRNA and protein levels of TXNIP and NLRP3; decreased levels of NLRP3 and ASC mRNA, and NLRP3, cleaved caspase-1, ASC and GSDMD-NT protein, released LDH and caspase-1 activity	cisplatin-induced ovarian damage model; LPS-treated human KGN cells	less decline in cisplatin-induced ovarian reserve, mitigation of cisplatin-induced follicular atresia, maintenance of AMH and E2 levels, improved cisplatin-induced ovarian fibrosis, restoration of normal estrous cycle, lower FSH levels, alleviation of cisplatin-induced acute ovarian injury	[128, 155]
Genipin and Tauro-ursodeoxycholic acid (TUDCA)	limit	decreases in mRNA levels of IL-1β, NLRP3, ASC and caspase-1, and protein levels of IL-18, IL-1β, NLRP3, caspase-1 and GSDMD-NT/GSDMD, and in levels of released LDH	KGN cells (testosterone/androgen-induced inflammation model)	performed only in vitro	[135]
Cysteine-rich 61 (CYR61)	limit	decreases in NLRP3, caspase 1, GSDMD and IL-1β protein levels	cyclophosphamide-induced rat GCs	higher viability of cyclophosphamide-treated rat GCs, decreased premature ovarian failure, increased number of mature and healthy follicles, decreased number of atretic follicles (control vs POI group)	[139]
Cyproterone Acetate (CYA)	limit	decreases in the protein levels of NLRP3, GSDMD, GSDMD-NT, caspase 1 and cleaved-caspase 1, decreases in IL-18 and IL-1β concentration in serum; activation of the IRE1 signaling pathway	mice GCs, PCOS mouse model induced by dehydroepiandrosterone (DHEA) (subcutaneous injection for 21 days), KGN cells	less cystic follicles, lower total testosterone	[150]

## Conclusion

Pyroptosis of ovarian granulosa cells is involved in the pathogenesis of multiple reproductive disorders including Premature Ovarian Insufficiency (POI) and Polycystic Ovary Syndrome (PCOS). Several factors, either endogenous / intrinsic or exogenous / extrinsic, have been identified so far, which have been shown to induce or inhibit / limit pyroptotic death of GCs, ultimately influencing fertility in females. Microplastics, di-(2-ethylhexyl) phthalate (DEHP), hyperandrogen (high levels of androgens), non-esterified fatty acids (NEFA), high-fat diet and letrozole, local glucose elevation have been reported to induce pyroptotic cell death in GCs, mostly mediated by NLRP3 inflammasome and GSDMD, in different in vitro and in vivo models. However, more research has focused on factors that inhibit or limit pyroptosis in GCs. Plumbagin, quercetin, HDAC1 (histone deacetylase 1), leonurine hydrochloride, certain Chinese traditional medicine recipes (Bushenhuoluo decoction (BSHLD), Bushen Cuyun recipe and He’s Yang Chao recipe), α-ketoglutarate, human bone marrow derived-mesenchymal stem cells (hBMSCs), metformin, genipin, tauro-ursodeoxycholic acid (TUDCA), cysteine-rich 61 (CYR61) and cyproterone acetate have been found to suppress pyroptotic death of GCs in different model systems of POI and PCOS. Similar to pyroptosis-inducers in GCs, these factors almost all the time influence NLPR3 inflammasome- and GSDMD-mediated pyroptotic cell death in GCs, decreasing pro-inflammatory death of these cells.

Combined, a better and more complete mechanistic understanding of pyroptosis-inducer and-inhibitor factors in granulosa cells is required to develop novel and more effective strategies against abnormal ovarian follicular development and associated reproductive disorders in women.

## Data Availability

Not applicable.
